# Role of IAPs in prostate cancer progression: immunohistochemical study in normal and pathological (benign hyperplastic, prostatic intraepithelial neoplasia and cancer) human prostate

**DOI:** 10.1186/1471-2407-10-18

**Published:** 2010-01-15

**Authors:** Gonzalo Rodríguez-Berriguete, Benito Fraile, Fermín R de Bethencourt, Angela Prieto-Folgado, Nahikari Bartolome, Claudia Nuñez, Bruna Prati, Pilar Martínez-Onsurbe, Gabriel Olmedilla, Ricardo Paniagua, Mar Royuela

**Affiliations:** 1 Department of Cell Biology and Genetics. University of Alcalá; 28871 Alcalá de Henares. Madrid, Spain; 2 Department of Pathology, Príncipe de Asturias Hospital. 28806 Alcalá de Henares. Madrid, Spain; 3 Department of Urology, "La Paz" Hospital. Madrid, Spain

## Abstract

**Background:**

In this study was investigate IAPs in normal human prostate (NP), benign prostatic hyperplasia (BPH), prostatic intraepithelial neoplasia (PIN) and prostatic carcinoma (PC), and their involvement in apoptosis/proliferation via NF-kB (TNF-α, IL-1) stimulation.

**Methods:**

Immunohistochemical and Western blot analyses were performed in 10 samples of normal prostates, 35 samples of BPH, 27 samples diagnosis of PIN (with low-grade PIN or high-grade PIN) and 95 samples of PC (with low, medium or high Gleason grades).

**Results:**

In NP, cytoplasm of epithelial cells were positive to c-IAP1/2 (80% of samples), c-IAP-2 (60%), ILP (20%), XIAP (20%); negative to NAIP and survivin. In BPH, epithelial cells were immunostained to c-IAP1/2 (57.57%), c-IAP-2 (57.57%), ILP (66.6%), NAIP (60.6%), XIAP (27.27%), survivin (9.1%). Whereas low-grade PIN showed intermediate results between NP and BPH; results in high-grade PIN were similar to those found in PC. In PC, epithelial cells were immunostained to c-IAP1/2, c-IAP-2, ILP, NAIP, XIAP (no Gleason variation) and survivin (increasing with Gleason).

**Conclusions:**

IAPs could be involved in prostate disorder (BPH, PIN and PC) development since might be provoke inhibition of apoptosis and subsequently cell proliferation. At the same time, different transduction pathway such as IL-1/NIK/NF-kB or TNF/NF-kB (NIK or p38) also promotes proliferation. Inhibitions of IAPs, IL-1α and TNFα might be a possible target for PC treatment since IAPs are the proteins that inhibited apoptosis (favour proliferation) and IL-1α and TNFα would affect all the transduction pathway involucrate in the activation of transcription factors related to survival or proliferation (NF-kB, Elk-1 or ATF-2).

## Background

Prostate cancers are generally slow-growing malignancies that are characterized by an imbalance in the rates of cell division and cell death [1]. Classical apoptosis is caused by the activation of caspases, a family of intracellular cysteine proteases that lie in a latent state in cells but become activated in response to a wide variety of cell death stimuli [2]. At the present, inhibitor of apoptosis proteins (IAPs) inhibits at least two of the major pathways for initiation of caspase activation: the mitochondrial pathway with cytochrome c; and the death receptor pathway with the tumour necrosis factor (TNF) family of death receptors [3,4]. TNF transduction pathway has been described by our group in previous manuscript [5,6].

IAPs is a gene family that plays an essential role in the negative regulation of apoptosis. IAPs family comprises eight proteins: Survivin, XIAP (ILP-1), c-IAP-1, c-IAP-2, NAIP, ILP-2, apollon (BRUCE) and ML-IAP (LIVIN).

c-IAP-1 and c-IAP-2 proteins are broadly expressed in normal human tissues with a cytoplasmic diffuse location pattern. Expression of c-IAP-1 and c-IAP-2 has been reported in several tumours such as in oesophageal [7], colon [8], uterine cervix [9] or prostate [10]. Several authors suggest that the expression of these proteins is associated with biological characteristic of cancer such as apoptosis [7] or aged-related [8].

The coding sequence of ILP-2 (IAP-like protein 2) [11] is very similar to that of XIAP (ILP-1), with 80% identity at the amino acid level [12]. Northern blot and RT-PCR analysis of both embryonic and adult human tissues reveals that ILP-2 expression is restricted to testis [13]. Overexpression of ILP-2 had no protective effect on death induced by bax or caspase 9 [11,12].

In human adult tissues, NAIP (neuronal apoptosis-inhibitory protein) transcripts were detected using Northern blot analysis in placenta, liver [14,15], spleen, lung [15], ovary or testis [16], with minimal expression in a number of other tissues, including foetal tissues [15]. By immunohistochemical analysis Maier et al. [16] described that the presence of NAIP in liver, lung and spleen is most likely due to macrophage infiltration. Choie et al. [17] described overexpression of NAIP in breast cancer patients with unfavourable clinical features, suggesting that NAIP would play a role in the disease manifestation. However, little is known about the clinical relevance of NAIP expression in breast cancer and in our knowledge no report described the presence in human prostate tissue.

Survivin is ubiquitously expressed in embryonic tissues where apoptosis occurs, but is not expressed or expressed at undetectable levels in several adult normal tissues. Overexpresion of survivin has been reported in several tumours such as in breast [16], colon [17], bladder [18], uterus [19], ovary [20], liver [21] or prostate [10,22]. Moreover, survivin has been reported to be expressed in some preneoplastic and/or benign lesions such as polyps of the colon, breast adenomas and PIN [10].

XIAP has been identified as one of the most potent inhibitor of caspases and apoptosis [23]. XIAP overexpression in tumour cells has been shown to cause an inhibitory effect on cell death besides to induce resistance to chemotherapy [23,24]. Equally to survivin, XIAP is overexpressed in precancerous PIN lesions [10].

Prostate diseases are chronic diseases that need a long development period. The evolution of PC needs a long period, probably due to the development of early and later precancerous modifications and then to the development of a clinical PC [25]. In this way, several clinical studies suggest that prostatic intraepithelial neoplasia (PIN) precedes carcinoma by 10 years or more, with low-grade PIN first emerging in men in the third decade of life [26,27].

In our knowledge, although some studies on c-IAP-1, c-IAP-2, XIAP and survivin have been reported, there are no reports on ILP-2 and NAIP (in prostate *in vivo *tissue), and studies on survivin are contradictory. The aim of the present study was to investigate by immunohistochemical methods these six IAPs family members in normal human prostate; their modifications in BPH, PIN and PC; and their possible involvement in apoptosis or proliferation via NF-kB (TNF-α and IL-1) stimulation.

## Methods

Prostates were obtained from: (a) transurethral resections from 33 men (aged from 53 to 88 years) with clinical and histopathological diagnosis of BPH; (b) radical prostatectomies from 27 men (aged from 20 to 59 years) diagnosis of low-grade PIN (12 men) or high-grade PIN (15 men); (c) radical prostatectomies from 95 men (aged from 54 to 69 years) with PC of low (Gleason scores < 6, 23 men), medium (Gleason scores 7, 51 men) and high (Gleason scores 8-10, 21 men) Gleason grades, with and without metastasis or lymph node infiltration at the time of surgery; and (d) histologically normal prostates (NP) obtained at autopsy (8-10 hours after death) from 20 men (aged from 20 to 38 years) without histories of reproductive, endocrine or related diseases. Each diagnosed sample was divided into two portions; one portion was immediately processed for immunohistochemistry, while the other portion that was frozen in liquid nitrogen and maintained at -80°C for Western Blotting analysis. In this later portion, cryostat sections were stained with toluidine blue to confirm histopathological diagnosis. Since PIN replace synonymous terms including intraductal dysplasia, marked atypia, hyperplasia with malignant changes or large acinal atypical hyperplasia, the reproductibility of the diagnosis of Low grade PIN is lack. All pathological, clinical or personal data were anonymized and separated from any personal identifiers. This study was made with the consent of the patients' relatives or their family in autopsy cases. All the procedures followed were examined and approved by the Principe de Asturias Hospital Ethics Committee (reference number SAF2007-61928) and were in accordance with the ethical standards of the Committee for Human Experimentation, with the Helsinki Declaration of 1975 (revised in 1983) and the Committee on Publication Ethics (COPE) guidelines.

The primary antibodies used were: rabbit polyclonal antibody XIAP and c-IAP-2; mouse monoclonal antibody survivin; goat polyclonal antibodies ILP-2, c-IAP-1/2 and NAIP (Santa Cruz Biotechnology, Ca, USA). For Western blot analysis [28,29] antibodies were diluted at 1:250 (survivin, NAIP, c-IAP-2, ILP), 1/500 (c-IAP-1/2, XIAP) and 1:10.000 chicken anti α-actin in TBS with 5% bovine serum albumin (BSA). For immunohistochemistry analysis [30,31] antibodies were diluted at 1:75 (survivin, c-IAP-2, ILP), 1:100 (NAIP), 1/150 (c-IAP-1/2, XIAP) in TBS.

Immunochemical procedure specificity was checked using negative and positive controls. For negative controls, tissues of each type (normal, BPH and PC) were incubated with blocking peptides (Sta. Cruz Biotechnology) or preimmune serum at the same immunoglobulin concentration used for each antibody. As positive controls, homogenates (for Western Blot) and histologic sections (for immunohistochemistry) of human skin, thymus or tonsils were incubated with the same antibodies.

A comparative histologic quantification of immunolabeling among the different types of prostates was performed for each antibody. From each prostate, six histologic sections were selected at random. In each section, the staining intensity (optic density) per unit surface area was measured with an automatic image analyzer (Motic Images Advanced version 3.2, Motic China Group Co., China) in 5 light microscopic fields per section, using the X20 objective. Delimitation of surface areas was carried out manually using the mouse of the image analyzer. For each positively immunostained section, one negative control section (the following in a series of consecutive sections) was also used, and the optic density of this control section was subtracted from that of the stained section.

From the average values obtained (by the automatic image analyzer) for each prostate, the means ± SD for each prostate type (NP, BPH, PIN and PC) were calculated. The results were corroborated by two different observers. The number of sections examined was determined by successive approaches to obtain the minimum number required to reach the lowest SD. The statistical significance between means of the different prostate group samples was assessed by the one way ANOVA test at p ≤ 0.05, by multiple pairwise comparisons (GraphPad PRISMA 3.0 computer program).

To determine whether the source of material (surgery or autopsy) could be responsible for changes in the immunohistochemical pattern, five prostatic biopsies (taken because of the suspicion of prostatic disease and their histologic study revealed a normal pattern) were processed for immunohistochemistry. The results of the quantitative immunohistochemical study in these biopsies were compared with those of autopsy prostates.

## Results

### Western blot analysis

**Figure 1:**
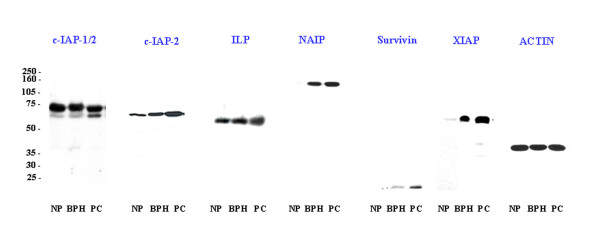
**Western blot analysis**. Western blot analysis of c-IAP-1/2, c-IAP-2, ILP, NAIP, Survivin, XIAP and α-actin after 15% polyacrilamide gel electrophoresis. NP: normal prostate. BPH: benign prostate hyperplasia. PC: prostate carcinoma. The lanes showing a band correspond to a positively stained prostate of each group.

Western blot analysis showed a single band for all the antibodies studied at the corresponding molecular weight in BPH and PC. In normal prostates (NP), only NAIP and survivin were not detected, while immunoreactions for the other antibodies were found at the corresponding molecular weight (figure 1).

### Immunohistochemistry

No immunoreaction was observed in the negative controls incubated with pre-immune serum, or using the antibodies preabsorbed with an excess of purified antigens.

No significant histologic or quantitative immunohistochemical differences between the two subgroups of normal prostates (biopsies and autopsies) were observed.

Positive immunostaining to c-IAP-1/2 was observed in the cytoplasm of epithelial cells in 80% of normal prostates patients, in 57.57% of patients with BPH, 59.25% of patients with PIN and in more 60% of PC patients (Table 1). Optic density to c-IAP-1/2 was similar in the four groups (NP, BPH, PIN and PC). In PC no differences were observed between the three Gleason groups.

**Table 1:** Percentages of patients showing positive immunohistochemical reactions to c-IAP-2, c-IAP-1/2 and ILP in normal prostate (NP), benign prostatic hyperplasia (BPH), prostatic carcinoma (PC) and average optical densities of immunostaining in positive patients.

	c-IAP-1/2		c-IAP-2		ILP	
**Prostates (no.)**	**Positive cases (%)**	**Optical density**	**Positive cases (%)**	**Optical density**	**Positive cases (%)**	**Optical density**

Normal (20)	80	19.64 ± 1.06	60	8.94 ± 0.61^a^	20	5.39 ± 1.18^a^

BPH (33)	57.57	18.36 ± 3.29	57.57	14.30 ± 1.2^b^	66.6	17.91 ± 1.44^b^

PIN						
Low grade PIN (12)	58.37	18.04 ± 1.29	66.6	9.83 ± 0.9^a^	66.6	7.26 ± 1.01^a^
High grade PIN (15)	60	18.91 ± 1.69	46.6	15.33 ± 1.52^b^	80	23.14 ± 1.49^c^

PC						
Low Gleason (23)	60.86	19.12 ± 1.63	39.13	14.44 ± 1.56^b^	82.6	23.87 ± 1.85^c^
Medium Gleason (51)	70.58	18.36 ± 2.38	39.21	19.08 ± 2.16^c^	82.35	23.79 ± 2.11^c^
High Gleason (21)	57.14	18.07 ± 1.94	42.85	18.45 ± 1.75^c^	100	27.78 ± 1.91^d^

Average optical densities were evaluated only in patients showing positive immunoreactions. Statistical analysis refers to each antibody separately. Values denoted by different superscripts are significantly different from each other. Significance was determined by the one way ANOVA test at p ≤ 0.05, by multiple pairwise comparisons (GraphPad PRISMA 3.0 computer program).

**Figure 2:**
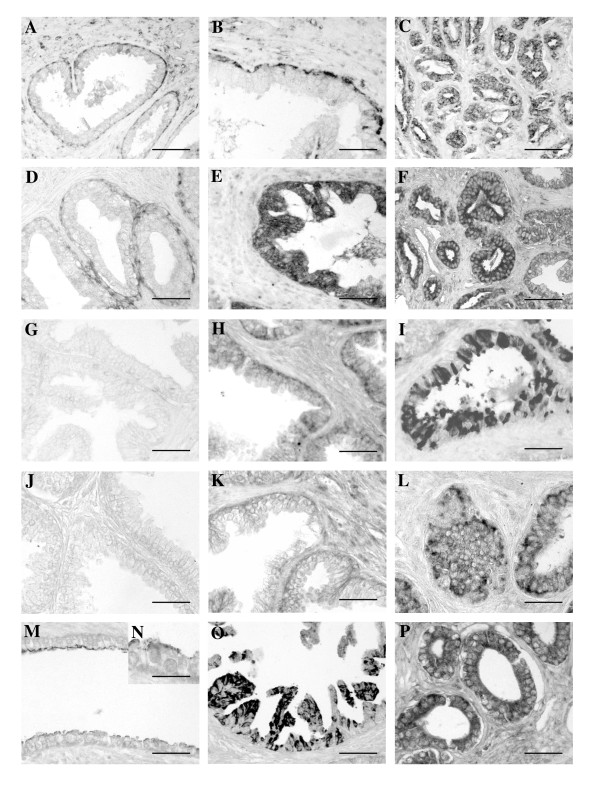
**Immunohistochemical analysis**. c-IAP-2 immunostaining appeared in the basal epithelial cells of normal (A) and BPH (B), whereas appeared in secretory epithelial cell of PC (C) samples. The cytoplasm of basal (normal and BPH) and secretory (cancer) epithelial cells presented positive immunoreaction to ILP in normal (D), high grade prostatic intraepithelial neoplasia (PIN) (E) and PC samples (F). No immunoreaction was found to NAIP in normal prostate (G) but was localized in the cytoplasm of epithelial cells in BPH (H) and PC (I) samples. Normal prostate (J) was negative to Survivin whereas immunoreaction was found in epithelial cells of BPH (K) and PC (L) samples. Immunoreaction to XIAP was observed in the apical cytoplasm of epithelial cells in BPH patients (Figs. M-N) whereas was perinuclear in high grade prostatic intraepithelial neoplasia (PIN) and PC (P) samples. Scale bars: 10 μm (O), 20 μm (B, E, H) and 30 μm (A, C-D, F-G, I-N, P).

Immunostaining to c-IAP-2 appeared in the cytoplasm of basal (normal and BPH) or secretory (cancer) epithelial cells in 60% of normal prostates (figure 2A), 57.57% of patients with BPH (figure 2B, Table 1), 55.15% of patients with PIN and in 40% of PC patients (figure 2C and Table 1). Optic density was higher in PC and BPH samples than in normal prostates. At the same time, similar optic density was observed in BPH and PC with low Gleason patients. The higher optic density was found in PC with medium and high Gleason, but not differences were observed between them.

ILP-2 always showed immunoreaction in the basal (normal, low grade PIN and BPH) or secretory (high grade PIN and cancer) epithelial cell cytoplasm in 20% of normal prostates (figure 2D, Table 1), 66.66% of BPH patients, 66.6% of low grade PIN, 80% of high grade PIN (figure 2E), 82.6% of cancer patients with low Gleason grade, 82.35% of cancer patients with medium Gleason grade (figure 2F), and all cancer patients with high Gleason grade (Table 1). Optic density was higher in PC samples than in normal prostates and BPH. In PC group, the higher optical density was observed in high Gleason group.

No immunoreaction to NAIP was found in normal prostate (figure 2G) and low grade PIN (Table 2). Positive immunoreaction was observed in the cytoplasm of epithelial cells in BPH (60.6% of patients) (figure 2H), high grade PIN (60%) and PC (more 58%) (figure 2I, Table 2). The highest optic density was found in PIN (high grade) and PC but no differences were observed between these or between three Gleason groups.

**Table 2:** Percentages of patients showing positive immunohistochemical reactions to NAIP, Survivin and XIAP in normal prostate (NP), benign prostatic hyperplasia (BPH), prostatic carcinoma (PC) average optical densities of immunostaining in positive patients.

	NAIP		SURVIVIN		XIAP	
**Prostates (no.)**	**Positive cases (%)**	**Optical density**	**Positive cases (%)**	**Optical density**	**Positive cases (%)**	**Optical density**

Normal (20)	0	^---^	0	---	20	6.96 ± 1.6^a^

BPH (35)	60.6	11.23 ± 2.9^a^	9.1	8.4 ± 1.51^a^	27.27	13.42 ± 0.59^b^

PIN						
Low grade PIN (12)	0	----	0	---	25	9.6 ± 1.21^c^
High grade PIN (15)	60	18.49 ± 2.02^b^	20	17.46 ± 1.57^b^	33.3	16.93 ± 1.73^d^

PC						
Low Gleason (21)	56.52	17.4 ± 1.96^b^	21.7	17.48 ± 1.53^b^	17.39	17.79 ± 1.55^d^
Medium Gleason (51)	58.82	18.25 ± 2.02^b^	13.72	17.66 ± 1.6^b^	39.21	16.64 ± 1.31^d^
High Gleason (21)	66.6	17.28 ± 2.46^b^	28.57	21.59 ± 1.35^c^	23.8	16.38 ± 1.03^d^

Average optical densities were evaluated only in patients showing positive immunoreactions. Statistical analysis refers to each antibody separately. Values denoted by different superscripts are significantly different from each other. Significance was determined by the one way ANOVA test at p ≤ 0.05, by multiple pairwise comparisons (GraphPad PRISMA 3.0 computer program).

Immunoreaction to survivin was absent in normal prostates (figure 2J) and low grade PIN (Table 2). Cytoplasmic immunoreaction of epithelial cells was observed in 9.1% of BPH (figure 2K), in 20% of high grade PIN and 19.35% of PC patients (figure 2L and Table 2). Optic density was higher in PC samples and high grade PIN. At the same time in PC groups the higher optical density was found in high Gleason group.

Immunoreaction to XIAP was observed in the apical cytoplasm of epithelial cells in 20% of normal prostates, 27.27% of BPH patients (figures 2M-N) and 25% of low grade PIN (Table 2). In high grade PIN (33.3%) (figure 2O) and PC (17.39% with low Gleason grade, 39.21% with medium Gleason grade (figure 2P, Table 2), and 23.8% with high Gleason grade) immunoreaction was perinuclear. Optic density was higher in BPH than in normal prostate and even higher in high grade PIN or PC, but no differences between PIN and PC Gleason groups were found.

All normal prostates and low grade PIN showed positive immunostaining to p-Elk-1, and this immunoreaction was observed in the nuclei of basal epithelial cells [31]. In BPH, the epithelial cells showed nuclear immunoreaction in 82.85% of patients and cytoplasmic immunoreaction in 14.28% of patients [31]. In high grade PIN, the epithelial cells showed nuclear immunoreaction in 40% of patients and cytoplasmic immunoreaction in 60% of patients. In PC, immunoreaction was nuclear in most of 33% of patients and cytoplasmic in most of 66.6% patients (Table 3). The highest optic density was detected in PC and High grade PIN samples (Table 3) [31].

**Table 3:** Percentages of patients showing positive immunohistochemical reactions to p-ELK-1, ATF-2 [31], p50 and p65 [5] in normal prostate (NP), benign prostatic hyperplasia (BPH) and prostatic carcinoma (PC) and average optical densities of immunostaining in positive patients.

	p-ELK-1		p-ATF-2		p50		p65	
**Prostates (no.)**	**Positive cases (%)**	**Optical density**	**Positive cases (%)**	**Optical density**	**Positive cases (%)**	**Optical density**	**Positive cases (%)**	**Optical density**

Normal (20)	100	2.41 ± 0.9^a^	100	3.34 ± 0.83^a^	60	7.88 ± 2.4*	0	

BPH (35)								
Cytoplasm	14.28	9.42 ± 5.13*			100	18.6 ± 1.72^#^	71.4	9.08 ± 3.23*
Nucleus	82.85	10.52 ± 2.56^b^	45.71	5.25 ± 2.16^a^				

PIN								
Low grade PIN (12)	100	3.22 ± 0.7^a^	100	4.32 ± 1.09*	58.3	15.32 ± 2.00^#^	0	
High grade PIN (15)								
Cytoplasm	60	17.35 ± 1.45^#^	53.3	13.35 ± 1.78^#^	86.6	27.51 ± 2.79^§^	93.3	30.17 ± 3.08^#^
Nucleus	40	18.27 ± 2.31^c^	26.6	12.31 ± 4.21^b^	26.6	28.76 ± 3.31^a^	13.3	29.77 ± 2.13^a^

PC								
Low Gleason (21)								
Cytoplasm	66.6	19.62 ± 5.64^#^	61.9	12.68 ± 3.28^#^	80.9	28.01 ± 2.25^§^	100	23.8 ± 2.93^§^
Nucleus	42.85	18.65 ± 3.4^c^	23.8	10.58 ± 1.2^b^	30.1	32.08 ± 2.96^a^	19	28.34 ± 2.04^a^
Medium Gleason (51)								
Cytoplasm	72.54	17.75 ± 4.54^#^	68.62	11.75 ± 3.45^#^	94.1	29.17 ± 1.22^§^	92.1	29.09 ± 1.90^#^
Nucleus	37.25	17.23 ± 3.56^c^	19.6	9.56 ± 3.87^b^	41.2	33.82 ± 4.07^a^	45.1	34.91 ± 4.32^b^
High Gleason (21)								
Cytoplasm	85.71	19.76 ± 4.78^#^	76.19	12.98 ± 5.11^#^	100	39.37 ± 4.15^¥^	80.9	36.71 ± 2.18^¥^
Nucleus	33.3	18.76 ± 3.87^c^	14.28	13.43 ± 3.56^b^	47.6	41.26 ± 2.31^b^	71.4	46.56 ± 1.20^c^

Average optical densities were evaluated only in patients showing positive immunoreactions. Statistical analysis refers to each antibody separately. Statistical analysis also separated cytoplasmic (symbol superscripts) and nuclear (letter superscripts) values. Values denoted by different superscripts are significantly different from each other. Significance was determined by the one way ANOVA test at p ≤ 0.05, by multiple pairwise comparisons (GraphPad PRISMA 3.0 computer program).

All normal prostates and low grade PIN showed immunoreaction to p-ATF-2 in the nuclei of basal epithelial cells (Table 3). In BPH, the immunoreaction was found in the nucleus of epithelial cells in 45.71% of patients [31]. In PC and high grade Gleason, immunostaining was nuclear in most of 14% of patients and cytoplasmic in most of 50% of patients (Table 3). The highest optic density was found in PC and high grade Gleason samples [31].

Scanty immunoreaction to NF-kB/p50 was localized in the cytoplasm of epithelial cells in 60% of normal prostate, 58.3% of low-grade PIN [5] and 100% of BPH patients. In PC, immunostaining was more intense (higher optic density) and appeared in the cytoplasm of epithelial cells in 80.9% of low Gleason grade, 4.1% of medium Gleason and 100% of high Gleason, and also in the nucleus of epithelial cells in 30.1% of patients with low Gleason, 41.2% with medium Gleason, and 47.6% of high Gleason (Table 3) [5]. Similar data were observed in high-grade PIN, where immunoreaction was found in cytoplasm (86.6% of patients) and nucleus (26.6% of patients) of epithelial cells (Table 3) [5].

No immunoreaction to NF-kB/p65 was observed in normal prostates and low-grade PIN samples [5]. In BPH immunoreaction appeared in cytoplasm of epithelial cells in 71.4% of cases. In PIN, cytoplasmic (93.3%) and nuclear (13.3%) immunostainings were found in epithelial cells. In PC cytoplasmic immunostaining was observed in 100% of low Gleason grade, 92.1% of medium Gleason and 80.9% of high Gleason, and nuclear immunostaining was found in 19% of low Gleason grade, 45.1% of medium Gleason and 71.4% of high Gleason (Table 3) [5]. Optical density was higher in PC and high-grade PIN patients than in BPH. No differences in optic densities regarding location (cytoplasm or nucleus) or Gleason grade were found (Table 3) [5].

## Discussion

The processes of both cell survival and cell death have involved highly regulated signalling pathways that are currently the subject of intense investigation. The rates of epithelial cell growth and death in adult normal prostate glands are in equilibrium [1]. The signalling pathways that lead to apoptosis or cell growth are beginning to be defined, and a number of proteins have been identified.

We used immunohistochemical analysis in order to know the percentage of positive patients, the location and the expression of each protein studied. So, the immunohistochemical analysis data are used to obtain the optical density data as we described in methods sections.

In normal prostate, we observed immunoexpressions to c-IAP-1/2, c-IAP-2, ILP-2, XIAP and NF-kB (p-50) in the cytoplasm of epithelial cells; p-Elk-1 and p-ATF-2 in the epithelial cell nuclei; but no immunoreaction to NAIP, survivin and NF-kB (p-65) were found (see Table 3). Several publications described survivin expression in normal tissues [26]. However, other authors have been reported that immunoexpression is not detected by immunohistochemistry in normal tissues [27] and has been only described by RT-PCR [25]. Vischioni et al. [28] also described cytoplasmic localization to c-IAP-1/2, c-IAP-2 and XIAP in normal prostate. The cytoplasmic localization described might be related to their binding to plasma membrane receptors such as the bone morphogenic protein type I receptors in the case of XIAP [32] or the tumour necrosis factor-receptor complexes in the case of c-IAP-1 and c-IAP-2. In low grade PIN we obtained similar results to those described in normal prostates samples. These observations could be suggest that expressions of these IAPs in normal prostate and PIN could be promoted the equilibrium between proliferation and apoptosis described in normal tissues. Since these IAPs were found in considerably amounts in BPH, high grade PIN and PC specimens, evaluation of these products can be helpful to discriminate between prostatic tissues that are normal and those subjected to pathologic proliferative processes.

In BPH, we detected immunoreactions to c-IAP-1/2, c-IAP-2, ILP-2, NAIP, survivin, XIAP, p-Elk-1, p-ATF-2 and NF-kB (p50 and p65). Similar results have been described by other authors to survivin [33]. These IAPs that are increased in BPH perhaps might be provoked the lower apoptosis index described in this benign pathology. This agrees with the high levels of other factor relate with proliferation or anti-apoptotic factors (such as NF-kB, bcl-2, mcl-1, p21 or Rb) and reported in the same patients used in this study [4,30,34]. De Miguel et al. [35] in the same samples used in this study, described that the proliferation index (measured by Ki-27 nuclear antigen) increased respect to that of the normal prostate, but the apoptotic index (measured by TUNEL) was similar than normal and, therefore, the equilibrium is displaced toward proliferation. These data could be suggests that an active stimulation of cell proliferation and survival occurs.

In PC, we described that the percentage of patients positives to all the IAPs family members studied is similar (c-IAP1/2, NAIP or XIAP), major (ILP-2, Survivin) or minor (c-IAP-2) than in BPH. NF-kB (p50 and p65) changes the location from the cytoplasm to the nucleus [5] whereas p-Elk-1 and p-ATF-2 changes the location from the nucleus to the cytoplasm in most of the PC patients examined [31]. The nuclear translocation of NF-kB activates target genes involved in carcinogenesis, such as IAPs [5]. Cytoplasm location to p-Elk-1 and p-ATF-2 could be mutant form that present altered functions and may be associated with the prostate cancer development [31]. In the other hand, optical density is more elevated in PC than in BPH (c-IAP-2, ILP, NAIP, Survivin, XIAP, NF-kB, p-Elk-1 and p-ATF-2); but only survivin and NF-kB increased with Gleason grade. We observed that immunoreactions were cytoplasmic (c-IAP-1/2, c-IAP-2, ILP, NAIP and survivin) or perinuclear (XIAP). The percentages of patients as well as the optical densities observed in high grade PIN have been similar to those observed in PC. These data also have been obtained by others authors. Similar localization to c-IAP-1, c-IAP-2 and XIAP in PC3 cells was described by McEleny et al. [36] in *in vitro *studies, suggesting that IAP may be an important contribution to apoptotic resistance in patients with prostate cancer. Krajewska et al. [10] by immunohistochemical analysis of prostate tumour tissues reveals cancer-specific elevations in the expression of cIAP1, cIAP2, survivin and XIAP; but immunostaining data did not correlate with Gleason scores or PSA. Vischioni et al. [28], also described cytoplasmic localization to XIAP. In several prostate cancer cell lines (PC3 and LNCaP), the expression of XIAP was perinuclear [36]. XIAP overexpression in tumour cells has been shown to cause an inhibitory effect on cell death induced by a variety of apoptotic stimuli [23]. In prostate adenocarcinoma, survivin has been shown to be overexpressed in an elevated proportion of patients. Nevertheless, results about correlations among survivin expression and both clinical and pathological features are contradictory [10,36-40]. An important finding is the involvement of survivin with resistance to antiandrogen therapy for prostate cancer [39]. At the present and in our knowledge, this is the first manuscript that describes ILP-2 and NAIP in human prostate *in vivo *tissue. In breast cancer survivin and NAIP overexpression has been associated in unfavourable clinical features [17]. In our data survivin (but not NAIP) only was increased in high Gleason grade. At the same time, we observed different localization to NAIP in cancer (compared to BPH) since not all epithelial cells were positives.

In general, IAPs have been shown to protect cells from a wide range of apoptotic triggers including Fas ligation, bax, activated caspases, cytochrome C, TNFα, some chemotherapeutic agents viral infection and radiation [36]. Elevations in the expression of several IAPs occur as a frequent and early event in the etiology of prostate cancer. Activation of NF-kB (translocation to the nucleus) promotes cell growth and proliferation in prostate cancer cells by regulating the expression of c-myc, cyclin D1 or IL-6 [29,40]; but also by the up-regulation of the expression of several anti-apoptotic proteins, including the inhibitor of apoptosis proteins (IAPs) [29]. Recent report [41,42] described IAPs family as downstream targets of activated NF-kB. In previous reports made in the same samples used here we described that in PC, the indexes of proliferation (by Ki-67 nuclear antigen and PCNA) and apoptosis (by TUNEL) were higher than in NP and BPH samples [35]. Nuñez et al. [5] using the same PC samples that we use in this study, also was observed increased levels of NF-kB, concluded that NF-kB is a new predictive marker of prostate cancer. At the same time activation to NF-kB together activation of other transcription factors, also studied in this patients by our group, such ATF-2 and Elk-1 [31] (related with enhanced cell proliferation and survival) might be due the overexpression of several components of the IL-1/NIK/NF-kB or TNF/NF-kB (NIK or p38) pathways [5]. At the same time activation of ATF-2, Elk-1 and NF-kB also are the consequence of p38 pathway (IL-1/p38 or TNF-p38) activation [31].

## Conclusions

In summary, it is reasonable to speculate that IAPs could be involved in prostate disorder (BPH, PIN and PC) development since might be provoke inhibition of apoptosis. So, the attempt of these proteins to respond against proliferation is insufficient. At the same time, different transduction pathway such as IL-1/NIK/NF-kB or TNF/NF-kB (NIK or p38) also promotes proliferation by activation of several transcription factors as NF-kB but also Elk-1 or ATF-2. Interpretation of these data suggests that, in prostate cancer, there are a higher number of factors relate with activation of mitogenic signaling cascades. In order to search a dominant target for therapy, it should be taken into account that PC is a heterogeneous disease in which multiple transduction pathways may interact in the uncontrolled apoptosis/cell proliferation. Inhibitions of IAPs, IL-1α and TNFα might be a possible target for PC treatment since IAPs are the proteins that inhibited apoptosis (favour proliferation) and IL-1α and TNFα would affect all the transduction pathway involucrate in the activation of transcription factors related to survival or proliferation (NF-kB, Elk-1 or ATF-2).

## List of abbreviations used

IAPS: inhibitory apoptosis proteins; ILP-2: IAP-like protein 2; NAIP: neuronal apoptosis-inhibitory protein; NP: normal prostate; BPH: benign prostatic hiperplasia; PIN: prostatic intraepithelial neoplasia; PC: prostatic carcinoma; IL: Interleukin; NF-kB: nuclear factor kappa B; NIK: NF-kB Inducing Kinase; PCNA: proliferating cell nuclear antigen; kDa: Kilodalton; MD: Medical doctor; COPE: Committee on Publication Ethics; M: Molar; mM: MiliMolar; SDS: sodium dodecyl sulphate; TBS: Tris Buffered Saline; BSA: bovine serum albumine; TBST: TBS/Tween-20; IHC: Immunohistochemistry; ABC: avidin-biotin-peroxidase complex; DAB: 3,3'-diaminobenzidine; TUNEL: terminal deoxynucleotidyl tranferase-mediated dUTP nick end-labeling method; SD: standard deviation; TNF: Tumor necrosis factor; TNFR: Tumor necrosis factor receptor.

## Competing interests

The authors declare that they have no competing interests.

## Authors' contributions

GR and MR designed the study and carried out the immunohistochemistry studies. BF, NB and CN participated in western blot analysis and result interpretation. PM, FB and GO prepared and provided the tumour biological samples and participated in the immunohistochemistry studies. BP, NB and AP performed Quantitative analysis and participated in discussion. FB and AP performed the statistical analysis and participated in the discussion. MR and RP participated in study coordination and supervision. All authors read, discussed and approved the final manuscript.

## Pre-publication history

The pre-publication history for this paper can be accessed here:

http://www.biomedcentral.com/1471-2407/10/18/prepub
